# Supplementary material for: Chart builder: an interactive tool for user driven data visualization in the electron microscopy data bank
Source: Front Bioinform. 2026 Mar 6;6:1763403. doi: 10.3389/fbinf.2026.1763403 (PMC13002805; doi:10.3389/fbinf.2026.1763403)
Supplement: Supplementary file 1 [file Table1.docx]

***Supplementary Material***

# Supplementary Figures and Tables

*Table S1: URL’s for up-to-date interactive views of all reported EMDB Chart Builder visualisations.*

| **Figure** | **Chart Builder URL** |
| --- | --- |
| Figure 1b | <https://www.ebi.ac.uk/emdb/statistics/builder?db=emdb&type=line&x_data=yearly&y_data=emdb_id&att_stack=unstacked&att_math=linear&x_field=&y_field=&z_field=&geo_field=grant_country_ft&y_label=Total+EMDB+entries&title=The+cumulative+growth+of+the+EMDB+archive&x_year1=2002&x_year2=2025&s_label1=EMDB&s_op1=cumulative&s_color1=%2338b248> |
| Figure 1c | <https://www.ebi.ac.uk/emdb/statistics/builder?db=empiar&type=line&x_data=yearly&y_data=empiar_id&att_stack=unstacked&att_math=linear&x_field=&y_field=&z_field=&geo_field=pi_country_code&y_label=Total+EMPIAR+entries&title=The+cumulative+growth+of+the+EMPIAR+archive&x_year1=2002&x_year2=2025&s_label1=EMPIAR&s_op1=cumulative&s_color1=%23990bda> |
| Figure 2a | <https://www.ebi.ac.uk/emdb/statistics/builder?db=emdb&type=area&x_data=yearly&y_data=emdb_id&att_stack=percent&att_math=linear&x_field=&y_field=&z_field=&geo_field=grant_country_ft&y_label=Proportion+%28%25%29+of+all+EMDB+entries&title=Entry+resolutions+in+the+EMDB+%28%C3%85%29&x_year1=2002&x_year2=2025&s_label1=%3C2&s_query1=resolution%3A%5B*+TO+2%7D&s_op1=cumulative&s_color1=%23210099&s_label2=2-3&s_query2=resolution%3A%5B2+TO+3%7D&s_op2=cumulative&s_color2=%23004cff&s_label3=3-4&s_query3=resolution%3A%5B3+TO+4%7D&s_op3=cumulative&s_color3=%237099ff&s_label4=4-5&s_query4=resolution%3A%5B4+TO+5%7D&s_op4=cumulative&s_color4=%233cfaea&s_label5=4-6&s_query5=resolution%3A%5B5+TO+6%7D&s_op5=cumulative&s_color5=%2386ff05&s_label6=6-8&s_query6=resolution%3A%5B6+TO+8%7D&s_op6=cumulative&s_color6=%23fff236&s_label7=8-10&s_query7=resolution%3A%5B8+TO+10%7D&s_op7=cumulative&s_color7=%23ffa442&s_label8=10-15&s_query8=resolution%3A%5B10+TO+15%7D&s_op8=cumulative&s_color8=%23f5544c&s_label9=%3E15&s_query9=resolution%3A%7B15+TO+*%5D&s_op9=cumulative&s_color9=%23ad0023> |
| Figure 3a | <https://www.ebi.ac.uk/emdb/statistics/builder?db=emdb&type=3dscatter&x_data=yearly&y_data=emdb_id&att_stack=unstacked&att_math=linear&x_field=resolution&y_field=average_qscore&z_field=qscore_relative_percentile_value&geo_field=grant_country_ft&x_label=Author+provided+resolution&y_label=Average+model-map+Q-score&min_x=0&max_x=20&min_y=-0.05&max_y=1&s_label1=Q-score+resolution+normalised&s_op1=unique&s_color1=%23ab31d8> |
| Figure 4a | <https://www.ebi.ac.uk/emdb/statistics/builder?db=emdb&type=histogram&x_data=yearly&y_data=emdb_id&att_stack=unstacked&att_math=linear&x_field=author_resolution&y_field=&z_field=&geo_field=grant_country_ft&gf=structure_determination_method%3A%22singleParticle%22&x_label=Author+provided+resolution&hist_start=0&hist_end=15&hist_gap=0.5&s_label1=Single+particle+analysis&s_op1=unique&s_color1=%2338b248> |
| Figure 4b | <https://www.ebi.ac.uk/emdb/statistics/builder?db=emdb&type=histogram&x_data=yearly&y_data=emdb_id&att_stack=unstacked&att_math=linear&x_field=author_resolution&y_field=&z_field=&geo_field=grant_country_ft&gf=structure_determination_method%3A%22subtomogramAveraging%22&x_label=Author+provided+resolution&hist_start=0&hist_end=15&hist_gap=0.5&s_label1=Subtomogram+averaging&s_op1=unique&s_color1=%23ea3961> |
| Figure 5 | <https://www.ebi.ac.uk/emdb/statistics/builder?db=emdb&type=venn&x_data=yearly&y_data=emdb_id&att_stack=unstacked&att_math=linear&x_field=&y_field=&z_field=&geo_field=grant_country_ft&s_label1=PDB&s_query1=fitted_pdbs%3A%5B*+TO+*%5D&s_op1=unique&s_color1=%234a5a6b&s_label2=AFDB&s_query2=xref_ALPHAFOLD%3A%5B*+TO+*%5D&s_op2=unique&s_color2=%235145f7&s_label3=EMPIAR&s_query3=xref_EMPIAR%3A%5B*+TO+*%5D&s_op3=unique&s_color3=%2348c256> |
| Figure 6a | <https://www.ebi.ac.uk/emdb/statistics/builder?db=emdb&type=geo&x_data=yearly&y_data=emdb_id&att_stack=unstacked&att_math=linear&x_field=author_resolution&y_field=&z_field=&geo_field=ca_continent_ft&x_label=Author+provided+resolution&s_label1=EMDB+entries&s_op1=unique&s_color1=%231251ce> |
| Figure 6b | <https://www.ebi.ac.uk/emdb/statistics/builder?db=emdb&type=geo&x_data=yearly&y_data=emdb_id&att_stack=unstacked&att_math=linear&x_field=author_resolution&y_field=&z_field=&geo_field=ca_institution_ft&gf=continent_name%3A%22North+America%22&x_label=Author+provided+resolution&s_label1=EMDB+entries&s_op1=unique&s_color1=%231251ce> |
| Figure 6c | <https://www.ebi.ac.uk/emdb/statistics/builder?db=emdb&type=geo&x_data=yearly&y_data=emdb_id&att_stack=unstacked&att_math=linear&x_field=author_resolution&y_field=&z_field=&geo_field=ca_institution_ft&gf=continent_name%3A%22Europe%22&x_label=Author+provided+resolution&s_label1=EMDB+entries&s_op1=unique&s_color1=%231251ce> |
| Figure 6d | <https://www.ebi.ac.uk/emdb/statistics/builder?db=emdb&type=geo&x_data=yearly&y_data=emdb_id&att_stack=unstacked&att_math=linear&x_field=author_resolution&y_field=&z_field=&geo_field=ca_institution_ft&gf=continent_name%3A%22Asia%22&x_label=Author+provided+resolution&s_label1=EMDB+entries+%28Asia%29&s_op1=unique&s_color1=%231251ce> |
| Figure 7 | <https://www.ebi.ac.uk/emdb/statistics/builder?db=emdb&type=column&x_data=custom&y_data=emdb_id&att_stack=normal&att_math=linear&x_field=&y_field=&z_field=&geo_field=grant_country_ft&x_label1=RELION&x_query1=image_reconstruction_software%3A%22relion%22&x_label2=CRYOSPARC&x_query2=image_reconstruction_software%3A%22cryosparc%22&x_label3=cisTEM+%28Frealign%29&x_query3=%28image_reconstruction_software%3A%22cistem%22+OR+image_reconstruction_software%3A%22frealign%22%29&x_label4=EMAN1%2F2&x_query4=%28image_reconstruction_software%3A%22EMAN1%22+OR+image_reconstruction_software%3A%22EMAN2%22%29&s_label1=Image+reconstruction+software+in+SPA&s_query1=structure_determination_method%3A%22singleParticle%22&s_op1=unique&s_color1=%232f7ed8> |
| Figure 8a | <https://www.ebi.ac.uk/emdb/statistics/builder?db=emdb&type=streamgraph&x_data=yearly&y_data=xref_PUBMED&att_stack=unstacked&att_math=linear&x_field=&y_field=&z_field=&geo_field=grant_country_ft&x_year1=2013&x_year2=2025&s_label1=NAT+COMMUN&s_query1=journal%3A%22nat+commun%22&s_op1=unique&s_color1=%232f7ed8&s_label2=NATURE&s_query2=journal%3A%22nature%22&s_op2=unique&s_color2=%230d233a&s_label3=PNAS&s_query3=journal%3A%22pnas%22&s_op3=unique&s_color3=%238bbc21&s_label4=NAT+STRUCT+MOL+BIOL&s_query4=journal%3A%22nat+struct+mol+biol%22&s_op4=unique&s_color4=%23910000&s_label5=SCIENCE&s_query5=journal%3A%22science%22&s_op5=unique&s_color5=%231aadce&s_label6=CELL&s_query6=journal%3A%22cell%22&s_op6=unique&s_color6=%23492970&s_label7=BIORXIV&s_query7=journal%3A%22biorxiv%22&s_op7=unique&s_color7=%23f28f43&s_label8=ELIFE&s_query8=journal%3A%22elife%22&s_op8=unique&s_color8=%2377a1e5&s_label9=MOL+CELL&s_query9=journal%3A%22mol+cell%22&s_op9=unique&s_color9=%23c42525&s_label10=SCI+ADV&s_query10=journal%3A%22sci+adv%22&s_op10=unique&s_color10=%23a6c96a&s_label11=STRUCTURE&s_query11=journal%3A%22structure%22&s_op11=unique&s_color11=%23000000&s_label12=CELL+REP&s_query12=journal%3A%22cell+rep%22&s_op12=unique&s_color12=%239fdfb2&s_label13=NUCLEIC+ACIDS+RES&s_query13=journal%3A%22nucleic+acids+res%22&s_op13=unique&s_color13=%23c59fdf&s_label14=EMBO+J&s_query14=journal%3A%22embo+j%22&s_op14=unique&s_color14=%23dfd79f&s_label15=J+VIROL&s_query15=journal%3A%22j+virol%22&s_op15=unique&s_color15=%239fd4df&s_label16=J+MOL+BIOL&s_query16=journal%3A%22j+mol+biol%22&s_op16=unique&s_color16=%23df9fc2&s_label17=CELL+RES&s_query17=journal%3A%22cell+res%22&s_op17=unique&s_color17=%23afdf9f&s_label18=COMMUN+BIOL&s_query18=journal%3A%22commun+biol%22&s_op18=unique&s_color18=%23a29fdf&s_label19=J+BIOL+CHEM&s_query19=journal%3A%22j+biol+chem%22&s_op19=unique&s_color19=%23dfb59f&s_label20=PLOS+PATHOG&s_query20=journal%3A%22plos+pathog%22&s_op20=unique&s_color20=%239fdfc7&s_label21=NAT+MICROBIOL&s_query21=journal%3A%22nat+microbiol%22&s_op21=unique&s_color21=%23da9fdf&s_label22=CELL+DISCOV&s_query22=journal%3A%22cell+discov%22&s_op22=unique&s_color22=%23d2df9f&s_label23=NAT+CHEM+BIOL&s_query23=journal%3A%22nat+chem+biol%22&s_op23=unique&s_color23=%239fbfdf&s_label24=J+STRUCT+BIOL&s_query24=journal%3A%22j+struct+biol%22&s_op24=unique&s_color24=%23df9fad&s_label25=SCI+REP&s_query25=journal%3A%22sci+rep%22&s_op25=unique&s_color25=%239fdfa5> |
| Figure 8b | <https://www.ebi.ac.uk/emdb/statistics/builder?db=emdb&type=line&x_data=yearly&y_data=assembly_molecular_weight&att_stack=unstacked&att_math=logarithmic&x_field=&y_field=&z_field=&geo_field=grant_country_ft&x_year1=2002&x_year2=2025&s_label1=Lowest&s_query1=assembly_molecular_weight%3A%7B0+TO+*%5D&s_op1=min&s_color1=%232f7ed8&s_label2=Average&s_query2=assembly_molecular_weight%3A%7B0+TO+*%5D&s_op2=avg&s_color2=%230d233a&s_label3=Highest&s_query3=assembly_molecular_weight%3A%7B0+TO+*%5D&s_op3=max&s_color3=%238bbc21> |
| Figure 9 | <https://www.ebi.ac.uk/emdb/statistics/builder?db=emdb&type=3dscatter&x_data=yearly&y_data=emdb_id&att_stack=unstacked&att_math=linear&x_field=average_ccmask_value&y_field=average_ccpeaks_value&z_field=qscore_relative_percentile_value&geo_field=grant_country_ft&x_label=CCmask+%28phenix+software%29&y_label=CCpeak+%28phenix+software%29&min_x=0&max_x=1.5&min_y=0&max_y=1&s_label1=Q-score+resolution+normalised&s_op1=unique&s_color1=%232f7ed8&s_label2=EMD-29721&s_query2=emdb_id%3AEMD-29721&s_op2=unique&s_color2=%23ff00ea&s_label3=EMD-23275&s_query3=emdb_id%3AEMD-23275&s_op3=unique&s_color3=%23ff00ea&s_label4=EMD-15028&s_query4=emdb_id%3AEMD-15028&s_op4=unique&s_color4=%236cf000&s_label5=EMD-22692&s_query5=emdb_id%3AEMD-22692&s_op5=unique&s_color5=%236cf000> |
